# Improving parenting skill through the strong families program in Thailand

**DOI:** 10.1186/s13690-025-01743-9

**Published:** 2025-10-16

**Authors:** Nida Buawangpong, Aala El-Khani, Apinun Aramrattana, Chalermkwan Chutima, Watjana Arunrangsi, Chiraporn Tantihachai, Chaisiri Angkurawaranon, Amalee McCoy, Kawinthip Rinpon, Nopakoon Nantsupawat, Karen Peters, Zin Ko Ko Lynn, Wadih Maalouf, Wichuda Jiraporncharoen

**Affiliations:** 1 Department of Family Medicine, Faculty of Medicine, Chiang Mai University, Chiang Mai, Thailand; 2 Global Health and Chronic Conditions Research Center, Chiang Mai University, Chiang Mai, Thailand; 3 Prevention, Treatment and Rehabilitation Section United Nations Office on Drugs and Crime, Vienna, Austria; 4 Division of Psychology and Mental Health, The University of Manchester, Manchester, M139PL UK; 5 Upstream Family & Community Learning Center, Chiang Mai, Thailand; 6 Drugs and Health Programme, United Nations Office on Drugs and Crime (ROSEAP), Bangkok, Thailand; 7 Chiang Mai juvenile vocational training center, Chiang Mai, Thailand

**Keywords:** Parenting skills, Positive parenting, Family health, Program evaluation

## Abstract

**Background:**

The Strong Families program has been successfully implemented in multiple low- and middle-income countries. However, Thailand has only recently begun exploring the program’s potential.

**Objectives:**

To assess the feasibility, indicative effects, and adaptability of the Strong Families program.

**Methods:**

This pilot project was conducted between June and July 2024 in Chiang Mai, Chiang Rai, and Mae Hong Son provinces in Thailand. Project trainers and family caregivers participating in the Strong Families program were recruited. Qualitative data were gathered prospectively through semi-structured interviews guided by the RE-AIM framework for project evaluation. Quantitative data were collected retrospectively, including pre- and post-training assessments using validated tools for the Strong Families program: the Strengths and Difficulties Questionnaire (SDQ), Parent and Family Adjustment Scale (PAFAS), and Child & Youth Resilience Measure-Revised Person Most Knowledgeable version (PMK-CYRM-R). Paired t-tests were conducted to indicate effects.

**Results:**

A total of 10 trainers from 8 sites participated, with a majority being female and a mean age of 35.20 ± 5.78 years. Additionally, 47 participants joined the Strong Families program and completed pre- and post-training assessments, most of whom were female, with a mean age of 39.17 ± 10.71 years. Preliminary findings from the pre- and post-training assessments revealed significant improvements in family dynamics and child resilience. Most trainers who participated in the Train-the-Trainer (ToT) program found the Strong Families program easy to implement in their areas by following the guidelines provided. Trainers reported that the program enhanced family relationships and improved mutual understanding among family members. However, challenges were identified, including language barriers and age group differences among participants. Some trainers noted that certain sentences in the materials were difficult to understand and translating them into local languages posed additional challenges. Furthermore, older children completed activities more quickly and experienced more boredom compared to younger children, leading to reduced cooperation. The words/activity adjustments were made to align behavior with cultural norms of the community, such as a pat on the shoulder instead of hugging. Each area plans to expand family and trainer networks, focusing on developing local volunteer trainers to ensure sustainability and continuity. Trainers need ongoing support and feedback from instructors to increase confidence, improve skills, and adapt effectively, especially when working with vulnerable families.

**Conclusion:**

The pilot project demonstrated the feasibility and preliminary effectiveness of the Strong Families program. It holds significant potential for scalability and expansion to other areas in the future.

**Clinical trial:**

Not applicable.

**Table:** 

**Text box 1. Contributions to the literature**
• This study is the first to evaluate the feasibility and preliminary effectiveness of the Strong Families program in Thailand using both qualitative and quantitative methods.
• It provides insight into program adaptability across diverse community settings, including high-risk families and protective care centers.
• The findings highlight key cultural and operational considerations necessary for localizing international parenting interventions in Southeast Asian contexts.
• The study offers practical guidance for sustainable implementation through community-led trainer development and cost-effective strategies.

## Introduction

Families worldwide face numerous challenges that affect their daily lives, including unemployment, difficulties with education, and environmental struggles [1]. These hardships are often compounded by additional factors such as migration, residing in refugee camps, or living in conflict or post-conflict zones [2]. In such high-stress environments, developing appropriate parenting skills becomes critical, as these skills profoundly influence various aspects of children’s health and development. Early childhood interactions predominantly occur within the family, and children may develop vulnerabilities if their parents or caregivers lack nurturing abilities, effective parenting skills, or face challenges such as poor health or financial instability [3, 4]. Strengthening family skills and competencies can provide vital reciprocal support for both parents and children [5].

In Thailand, parenting practices reflect a blend of traditional cultural values, health-related challenges, and the pressures of globalization [6]. These interconnected factors create a unique parenting environment, requiring caregivers to adapt their approaches to effectively support their children [7]. Addressing communication barriers and socio-economic issues is also essential for fostering effective parenting practices [8]. However, Thailand’s rapidly changing social dynamics have introduced significant challenges, particularly in addressing youth behavior and domestic violence [9]. This is evidenced by rising rates of substance abuse and gaming addiction among adolescents [10, 11]. Moreover, over 10% of children aged 4 to 6 years are at risk of behavioral and emotional problems. Children exposed to physical, emotional, or sexual abuse face a heightened likelihood of developmental delays and reduced academic performance. In cases of multiple forms of violence, the risk of common mental health disorders increases by 4.6 times [12]. Research underscores the importance of effective parenting skills in mitigating these challenges, as they promote better communication, emotional intelligence, and stronger family relationships [13, 14].

To address these concerns, the United Nations Office on Drugs and Crime (UNODC) has been implementing evidence-based family skills prevention programs in low- and middle-income countries since 2010 [15]. In 2017, the Strong Families program was introduced, specifically designed for families in challenging settings. This evidence-informed initiative aims to enhance family skills to promote the health and safe development of children of both sexes. The program is adaptable to various contexts and helps families manage daily stress and difficulties. By strengthening family structures and functions, it seeks to prevent drug use, violence, and other negative social outcomes among children. The Strong Families program targets families with children aged 8 to 15 years, providing universal benefits within this selective subgroup [16].

The Strong Families program has been successfully implemented in numerous countries, improving family relationships and parenting skills. Originally developed and tested in Afghanistan, it has since expanded to Central America, Central and West Asia, and East and West Africa. Thailand has recently begun exploring the program’s potential at the community level. Therefore, the objective of this study is to assess a pilot Strong Families program in the Northern region of Thailand using the RE-AIM program evaluation framework in order to assess its feasibility, preliminary effectiveness, and adaptability.

## Methods

### Study type

A retrospective quantitative analysis of pre- and post-intervention data, and a prospective qualitative study involving interviews.

### The strong families program

This pilot project was conducted between June and July 2024 in Chiang Mai, Chiang Rai, and Mae Hong Son provinces, encompassing eight locations in total. Training for the Trainer (ToT) of the Strong Families program was a 3-day workshop for trainers who would conduct the Strong Families program in their local areas. The program facilitators were master trainers certified by UNODC. The Strong Families program consisted of three sessions held over three consecutive weeks. The first week is only for caregivers. The second and third weeks are for caregivers and their children, running parallel caregiver and child sessions separately, followed by a joint family session (caregiver and child together). The topics for caregivers included (1) Understanding strengths and stresses, (2) Using love and limits, and (3) Teaching children what is right. The topics for children included (1) Learning about stress and (2) Following rules and appreciating parents. The topics for joint sessions included (1) Learning about each other and (2) Supporting values and dreams [17]. Program facilitators followed and provided feedback for the trainers in community sites at the first session of the Strong Families program. In this study, the volunteers who participated were the trainers from each training location.

### Study population

The study purposively recruited two groups of participants involved in the Strong Families program: [1] volunteer trainers and [2] family members or caregivers who participated in the program.

For the prospective qualitative study, a total of 10 trainers (out of 30) from 8 sites across Northern Thailand were included. These trainers were Thai staff affiliated with the UNODC and had completed the Training of Trainers (ToT) course. Eligibility criteria for inclusion in the qualitative study included having completed the ToT program, being actively involved in program delivery, and being at least 20 years of age. Individuals were excluded if they were unable to communicate in Thai or unable to attend the full 7-week duration of the program.

For the retrospective quantitative study, the inclusion criteria required family members who had children aged 8–15 years, completed the Strong Families program, and were at least 20 years old.

### Sample size calculation

#### Prospective qualitative study

There were a total of 30 trainers across eight locations. This pilot project in Thailand includes a sample size of 10 participants, approximately one to two trainers per site. Volunteer trainers from each site were invited to be included. We would use data saturation to reach an adequate sample size. The previous systematic review suggested that 9–17 interviews or 4–8 focus group discussions could reach saturation [18].

#### Retrospective quantitative study

Using data from the first Strong Families program in Afghanistan [16], the program reported the total strengths and difficulty scale, which is the standard tool measured, as 11.55 ± 5.64 after completion. The sample size calculation is based on a finite population mean [17]. The required sample size is at least 24 participants to achieve the power of 80% to detect the effectiveness of the Strong Families program.

### Data collection

The qualitative interview was conducted after 6 weeks of post-training. Participant age and gender were collected as part of the demographic information. Qualitative data were gathered prospectively through semi-structured interviews guided by the RE-AIM framework [19, 20], assessing reach, effectiveness, adoption, implementation, and maintenance. Interviews with volunteer trainers were conducted as focus group interviews (everyone together) by the training location. Due to the potential influence of hierarchical dynamics, the facilitator was not involved in the interview to minimize bias. All participants were informed of the research study and provided consent with permission for audio recording. The interviews were conducted by researchers that were not involved in the program. Each interview lasted around 40 min and was conducted onsite. The interviews were transcribed verbatim. Researchers reviewed interview notes and listened to the recordings at the end of each session to monitor for thematic saturation. Saturation was considered achieved when no new themes emerged from the data [21].

**Fig. 1:**
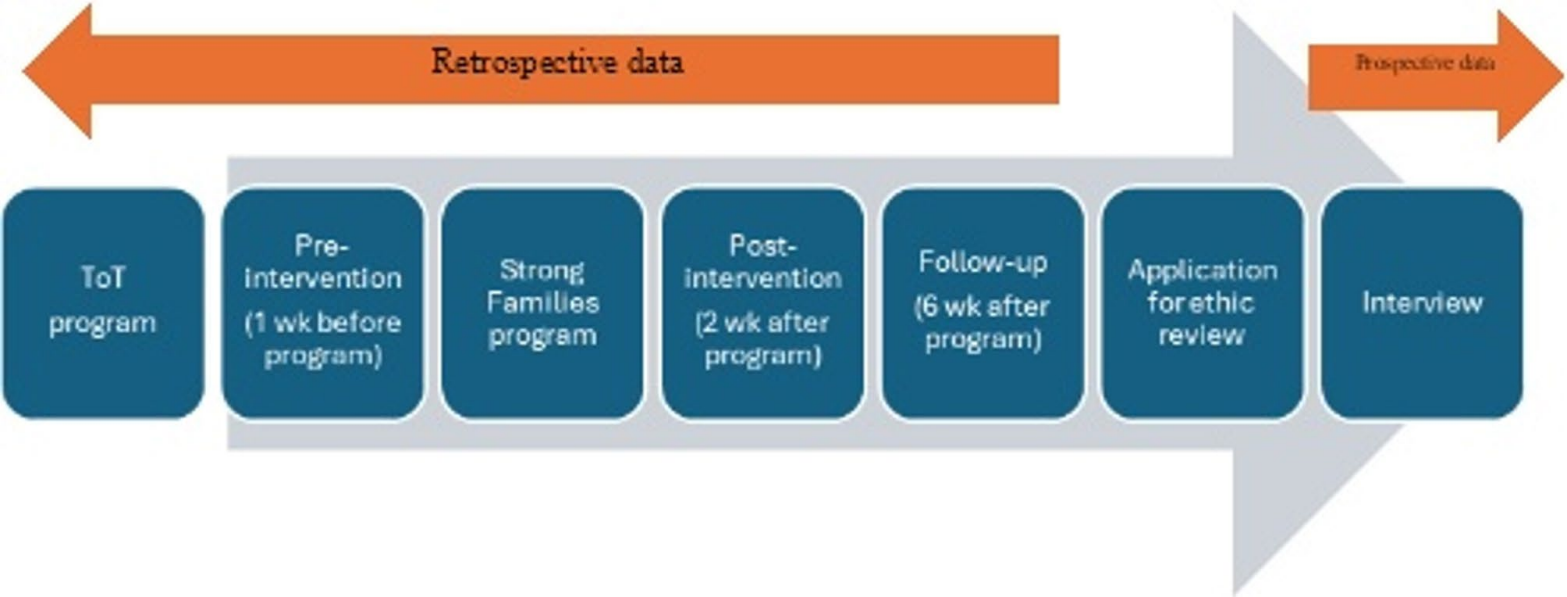
The timeline of the Strong Families program and research protocol

Quantitative data was collected retrospectively at 2 weeks and 6 weeks after program completion according to the guideline of program measurement [16], including participants’ age and gender and various validated tools for the Strong Families program: the Parent and Family Adjustment Scale (PAFAS) to reflect parents’/caregivers’ perspective on themselves and the Strengths and Difficulties Questionnaire (SDQ) and Child & Youth Resilience Measure-Revised Person Most Knowledgeable version (PMK-CYRM-R) to reflect parents’/caregivers’ perspective on the children. A timeline of the program and research protocol is detailed in Fig. 1.

The PAFAS has demonstrated good internal consistency among Australian samples, with Cronbach’s alpha values ranging from 0.70 to 0.87 [22]. It has also been shown to be applicable in other countries, including China [23], Panama [24], and Myanmar [25]. The SDQ has been used in previous studies conducted in Afghanistan [16, 26] and Myanmar [25], and has been validated in the Thai context with good reliability and internal consistency, with Cronbach’s alpha values of 0.63 [27, 28]. Similarly, the PMK-CYRM-R has been utilized in studies in both Afghanistan [16, 26] and Myanmar [25]. The internal consistency of the PAFAS, SDQ, and PMK-CYRM-R for this sample was 0.85–0.93, 0.86–0.95, and 0.89–0.97, respectively.

### Data analysis

For qualitative analysis, the interviews were recorded and transcribed verbatim and analyzed using thematic analysis [29]. The codes and themes were conducted regarding the RE-AIM framework [30]; Reach, Effectiveness or efficacy, Adoption, Implementation, and Maintenance in which Reach focuses on accessibility and recruitment strategies; Effectiveness focuses on the perceived impact of the program; Adoption focuses on staff and organizational readiness; Implementation focuses on fidelity, feasibility, cost, and adaptation; and Maintenance focuses on sustainability factors. All transcripts were then coded by researchers (NB, WJ, and KR) using NVivo version 12. Codes were then developed based on patterns in the data. The initial codes were developed by three researchers (NB, WJ, and KR) and compared to ensure reliability. The identified codes were compared and discussed for similarities and differences until a consensus was reached on the emergent themes. The codes that emerged from the interviews were processed regarding the questions of the RE-AIM descriptions. The final codes and themes were confirmed by all researchers.

For quantitative analysis, descriptive statistics and paired t-tests were used to evaluate program preliminary effectiveness. The scoring key for PAFAS indicates that higher scores reflect lower levels of parenting skills and family adjustment [22]. For the SDQ, higher scores indicate greater problems in emotional regulation, conduct, restlessness, and peer relationships; however, they also indicate greater strengths in the social relationships domain [31]. For the PMK-CYRM-R, higher scores reflect higher levels of child and youth resilience [32]. The mean difference (MD) and 95% confidence interval (CI) were analyzed using a paired t-test to compare the measurement scores between pre-training and 6 weeks post-training. All statistical analysis was conducted using STATA version 16 (Stata Corp, College Station, Texas). The p-value of less than 0.05 was indicated to identify statistical significance.

## Results

There were overall 10 trainers from 8 sites. The majority of them were female (60.0%) with a mean age of 39.0 ± 11.7 years old. There were 47 parents/caregivers who joined the Strong Families program and responded to the pre- and post-training assessment. Most of them were female (59.1%) and the mean age of participants was 39.2 ± 10.7 years old.

**RE-AIM (Reach**,** Effectiveness or Efficacy**,** Adoption**,** Implementation**,** Maintenance)**.

## Reach

### Theme 1.1 targets families in routine community activities

The target groups that trainers can easily access are those who engage in activities with community organizations, such as religious groups. For example, tribal groups that participate through religious mechanisms, such as families attending church every Sunday, tend to maintain good relationships. However, in some areas where literacy is limited, reaching the target audience requires verbal communication to ensure understanding and encourage participation in the program.

*“I think it’s an advantage for us because we already have a project working with families. This means we have a good relationship with them*,* as we work through the church. This makes building relationships easier*,* and families respond and cooperate well*,* even if we don’t know them directly*,*” A01.*

*“A limitation in inviting vulnerable families is illiteracy or work obligations. To address this*,* we made agreements with families to bring their children to the program*,* requiring their cooperation and providing information on why they should participate. A good relationship with the families is key*,*” A02.*

### Theme 1.2 Hard-to-reach groups May be easy to reach in certain locations

Families facing psychological issues, substance abuse, or domestic violence are harder to access and do not participate in activities. However, some areas reported easier access to families of substance users due to ongoing collaboration with drug rehabilitation centers.

*“Engaging high-risk groups in activities is challenging. Families with issues*,* especially those involving gambling or substance abuse*,* often lack focus and are difficult to inform. While other families participating in the program tend to have a willingness to improve their situation*,* however*,* substance-abusing families usually drop out early*,*” A01.*

*“We are the center works with rehabilitation for substance-used cases. In our area*,* most participants are families with drug-related problems. They also invite other families with drug related issue to join the program*,*” B04.*

In closed environments, such as protective care and welfare centers, children and caregivers can directly participate in activities, with caregivers representing parents due to limitations in parental involvement.

*“This program teaches family skills*,* communication*,* interaction*,* and activities with children. We initially train caregivers because children currently live with them in protective and welfare centers. Caregivers select up to two children they have a close relationship with. We haven’t reached the families directly since the children stay with us*,* and their parents are far away*,*” C05.*

*“The foundation provides care for orphans or children without guardians and those at risk of trafficking. Our protective care program targets children aged 11–18 and their caregivers rather than their real families due to the challenges of parental availability over the three-week program*,*” D07.*

## Effectiveness

### Theme 2.1 improved parent-child relationships, communication, and stress management

Trainers reflected on the effectiveness of family-strengthening activities, emphasizing improved communication, positive interaction, and reduced emotional problems for both parents and children. Activities such as stress management also contributed to happier families. However, some areas reported less favorable outcomes, due to previous relationships between children and caregivers in welfare centers.

*“One parent thanked us for involving them. Simple things like praising their children were new to them. Through the program*,* they learned positive communication and saw changes in their children*,*” A03.*

*“The group therapy-like activities allowed participants to share and express themselves. Children and caregivers understood each other better*,* improving their relationship. Some children who had never expressed love or gratitude were able to do so through activities*,*” C06.*

*“Stress management for parents resulted in better child behavior and reduced emotional problems*,* leading to happier families*,*” B04.*

*“Families appreciated the training*,* even if they didn’t fully understand everything*,* and tried to improve themselves. For stressed parents*,* simple breathing exercises were helpful*,*” E08.*

*“Pairing children with caregivers had mixed results. Some pairs worked well and had fun*,* while others*,* lacking familiarity*,* showed less enthusiasm. It depended on the individual relationship*,*” D07.*

**Fig. 2:**
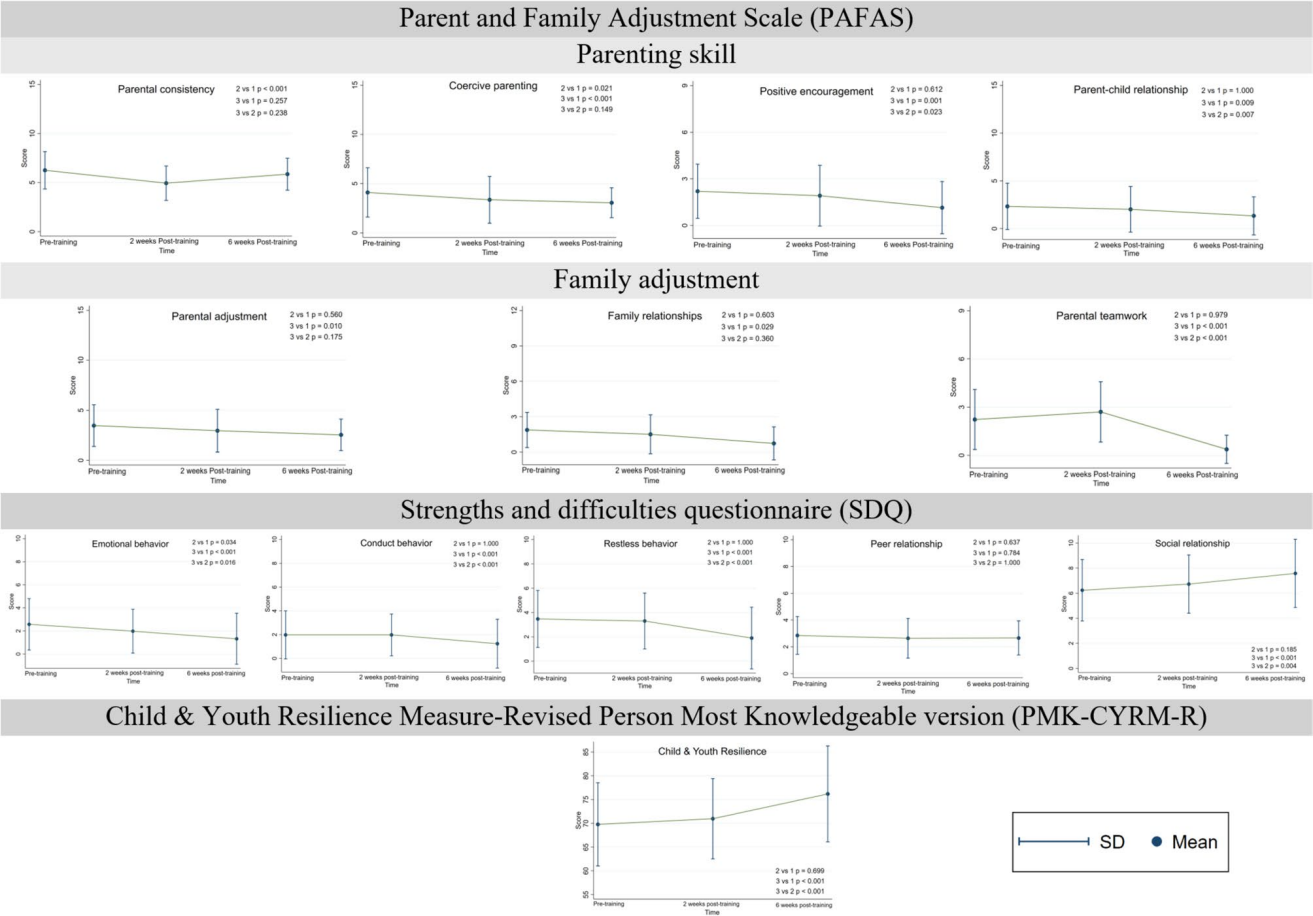
the results of all assessment tools on pre- and post-training

The preliminary indicative effects of the Strong Families program were also evaluated using standard tools. Figure 2 illustrates the results of all assessment tools on pre-training, 2 weeks post-training, and 6 weeks post-training. The mean scores and standard deviations are presented in Table 1.

#### Parent and family adjustments (PAFAS)

Most domains in PAFAS significantly improved. Coercive parenting improved over time (from 5.03 (SD 2.46) at pre-training to 3.14 (SD 1.62) in 6-week post-training, with MD -1.93 (95% CI -3.02 to -0.84, *p* = 0.001)), indicating that parents became more stable and predictable in their parenting approach. Positive Encouragement scores decreased (from 2.07 (SD 1.81) at pre-training to 1.21 (SD 1.63) in 6-week post-training, with MD -0.86 (95% CI -1.35 to -0.38, *p* = 0.001)), showing parents became more supportive. Parent-child relationships strengthened (from 2.48 (SD 2.25) at pre-training to 1.45 (SD 2.01) in 6-week post-training, with MD -1.03 (95% CI -1.86 to -0.21, *p* = 0.016)), with lower scores each session, suggesting better family bonding. Family adjustment showed improvement in parental adjustment (from 3.86 (SD 1.87) at pre-training to 2.62 (SD 1.71) in 6-week post-training, with MD -1.24 (95%CI -2.10 to -0.38, *p* = 0.006)), family relationship (from 1.72 (SD 1.33) at pre-training to 0.90 (SD 1.50) in 6-week post-training, with MD -0.83 (95%CI -1.50 to -0.15, *p* = 0.018)), and parental teamwork (from 2.28 (SD 1.62) at pre-training to 0.45 (SD 0.18) in 6-week post-training, with MD -1.89 (95% CI -2.66 to -1.13, *p* < 0.001)), reflecting better cooperation between family members. However, the results of parental consistency improved only during the 2-week post-training.

#### Emotional and behavioral outcomes (SDQ)

In children from parents’ perspective, emotional behavior, conduct behavior, and restless behavior declined from pre-training scores of 2.57 (SD 2.22), 1.98 (SD 2.03), and 3.47 (SD 2.35) to post-training scores of 1.32 (SD 2.21), 1.23 (SD 2.06), and 1.89 (SD 2.55) at the 6-week mark, respectively. The mean difference was − 1.26 (95% CI -1.73 to -0.78, *p* < 0.001), -0.74 (95% CI -1.13 to -0.36, *p* < 0.001), and − 1.57 (95% CI -2.30 to -0.85, *p* < 0.001), respectively, reflecting enhanced emotional regulation. Peer relationship gains were modest. Social relationships were improved due to a significant increase, MD 1.34 (95% CI 0.80 to 1.89, *p* < 0.001).

#### Child and youth resilience

Scores slightly increased across sessions, suggesting improved resilience among children (from 69.79 (SD 8.75) at pre-training to 76.19 (SD 10.09) in 6-week post-training). The mean difference was 6.40 (95% CI 4.13 to 8.68, *p* < 0.001).

**Table 1:** Mean scores and standard deviations of all assessment tools

Domains (*N* = 47)	Pre-training		2 weeks post-training		6 weeks post-training		Mean difference pre-training and 6 weeks post-training		
	Mean	SD	Mean	SD	Mean	SD	MD	95%CI	*p*-value
**Parent and Family Adjustment Scale (PAFAS)**									
**PAFAS Parenting skill**									
Parental consistency	5.97	1.64	4.91	1.76	5.72	1.69	-0.18	-1.17 to 0.68	0.597
Coercive parenting	5.03	2.46	4.43	2.40	3.14	1.62	-1.93	-3.02 to -0.84	0.001
Positive Encouragement	2.07	1.81	1.89	1.97	1.21	1.63	-0.86	-1.35 to -0.38	0.001
Parent-child relationship	2.48	2.25	1.96	2.38	1.45	2.01	-1.03	-1.86 to -0.21	0.016
PAFAS Family adjustment									
Parental adjustment	3.86	1.87	2.96	2.16	2.62	1.71	-1.24	-2.10 to -0.38	0.006
Family relationships	1.72	1.33	1.50	1.67	0.90	1.50	-0.83	-1.50 to -0.15	0.018
Parental teamwork	2.28	1.62	2.24	1.68	0.45	0.18	-1.89	-2.66 to -1.13	< 0.001
Strengths and difficulties questionnaire (SDQ)									
Emotional behavior	2.57	2.22	1.98	1.89	1.32	2.21	-1.26	-1.73 to -0.78	< 0.001
Conduct behavior	1.98	2.03	1.98	1.76	1.23	2.06	-0.74	-1.13 to -0.36	< 0.001
Restless behavior	3.47	2.35	3.30	2.30	1.89	2.55	-1.57	-2.30 to -0.85	< 0.001
Peer relationship	2.85	1.41	2.64	1.48	2.66	1.27	-0.19	-0.50 to 0.12	0.220
Social relationship	6.23	2.45	6.72	2.32	7.57	2.72	1.34	0.80 to 1.89	< 0.001
Child & Youth Resilience Measure-Revised Person Most Knowledgeable version (PMK-CYRM-R)									
Child and Youth Resilience	69.79	8.75	70.98	8.46	76.19	10.09	6.40	4.13 to 8.68	< 0.001

**Abbreviation**: confidence interval, CI; mean difference, MD; standard deviation, SD

## Adoption

### Theme 3.1 adopted by community organizations responsible for family health

The program was implemented by NGOs working with tribes through religious organizations, protective care centers, and the Ministry of Social Development and Human Security, aiming to improve child care quality, parenting skills, and family relationships.

*“We saw the program’s benefits and adapted it for caregivers acting as parents*,* achieving moderate success*,*” C05.*

*“We aim to apply the program across all tribes uniformly*,*” A03.*

*“We introduced the curriculum to community churches and organizations. Community leaders*,* particularly women leaders*,* coordinated the activities*,* which were well-received*,* with communities even helping identify participants*,*” E08.*

## Implementation

### Theme 4.1 fidelity to protocol amid operational challenges

Most participants followed the program correctly, guided by facilitators. However, challenges such as time management, language translation, and understanding of materials affected implementation.

*“Managing time was difficult as everyone wanted to share. Interrupting them felt wrong because it gave them an outlet*,*” C06.*

*“Some activities exceeded planned time due to discussions and local language translation*,*” A03.*

### Theme 4.2 feasible materials with translation and visual clarity challenges

Most participants found the manual and teaching materials straightforward and easy to use. The content of the activities was clear, with detailed steps, suggested dialogue, and required equipment well explained. The continuity of the weekly content also facilitated usability. The program’s hands-on approach made it practical for all age groups, even for illiterate families. However, some participants noted that the sequence of content could be confusing due to cross-referencing arrows, requiring additional time to understand. Furthermore, translations in some parts of the manual were hard to comprehend, seemingly translated from English, and further translating them into local languages posed challenges. Visual aids in the manual were sometimes unrealistic, hard to interpret, or unappealing to learners, such as facial expressions and coloring cards. Additionally, the font size was too small, especially for those with vision impairments.

*“The strength of this program is its simplicity. Anyone can use it. It provides clear instructions*,* images*,* and required equipment. With a bit of preparation*,* anyone can be a facilitator. It’s accessible to the general public*,* not just government agencies. The program fosters a sense of collaboration and mutual learning*,* making it engaging and approachable*,*” F09.*

*“The content is simple and not excessive. Learning is hands-on*,* immediately applicable*,* and suitable for parents of all ages. Even illiterate families can participate because of the practical focus. Children also engage well*,* such as by helping with household tasks*,*” A01.*

*“The language in some parts of the manual is difficult to understand*,* as it seems to have been translated from English. Translating it into local languages adds complexity*,* especially given the diverse dialects of the ethnic groups*,*” G10.*

*“The illustrations in the manual*,* like cartoon faces*,* could be more appealing to children. Bright and vibrant colors might help*,*” B04.*

*“There should be clearer illustrations accompanying the activities described in the manual to help facilitators better understand. Some activities are listed by name only*,* making it unclear how to execute them*,*” A01.*

### Theme 4.3 low-cost implementation with minimal expenses and potential for further cost reduction

Most areas reported low costs for organizing activities, primarily limited to food and drinks. Additional expenses included transportation and accommodation for trainers traveling to remote communities.

*“The activities are manageable with minimal investment or special equipment. If local staff are trained to continue the program*,* it could reduce travel and accommodation expenses. Since families are usually available only in the evenings*,* we provide dinner instead of financial compensation to encourage participation*,*” A01.*

*“Expenses are minimal if there are no refreshments. Families participate voluntarily*,* so there’s no need for compensation*,*” B04.*

### Theme 4.4 cultural adaptation through aligning methods and tools with local values and beliefs

Participants adapted their behavior and methods to align with the values and beliefs of parents and children. For instance, instead of hugging, facilitators used shoulder pats to respect cultural norms. Visual tools were adjusted, replacing cartoon faces with real human expressions for better clarity. Religious elements, such as prayers, were incorporated to help participants find inner peace.

*“The manual provides scripts for silent participants*,* but they don’t always fit local contexts. For example*,* parenting styles differ among ethnic groups*,* requiring improvisation*,*” B04.*

*“Our foundation works with caregivers rather than parents. Instead of hugs*,* we use shoulder pats to respect personal boundaries*,*” D07.*

*“For adults*,* facial expressions in the manual are ineffective. I found acting out the emotions directly worked better*,*” B04.*

*“Incorporating religious practices like prayer helps families calm their minds*,*” A03.*

## Maintenance

### Theme 5.1 local trainer development for sustainability

All areas aimed to expand the program by developing local trainer teams to ensure sustainability. Emphasis was placed on building strong relationships between trainers and families to foster cooperation.

*“Key motivators for continued participation include relationships between trainers and families*,* voluntary participation*,* and perceived value in the information provided*,*” A01.*

*“To ensure sustainability*,* capable local trainers should be developed. Community Development Centers and existing funding could support these efforts*,*” F09.*

*“For remote areas*,* consecutive three-day programs may be more feasible to address travel challenges*,*” E08.*

### Theme 5.2 support and skill development for long-term impact

Trainers emphasized the need for support, skill-building, and clear guidelines for long-term impact. Challenges included working with vulnerable families and addressing emotional situations.

*“Online training isn’t effective. Hands-on practice is essential*,*” A01.*

*“New trainers need feedback and support. Regular follow-ups during the first week and accessible expert advice would help*,*” A02.*

*“We need clearer follow-up tools to measure the program’s impact on family behavior and emotions over time*,*” C05.*

**Fig. 3:**
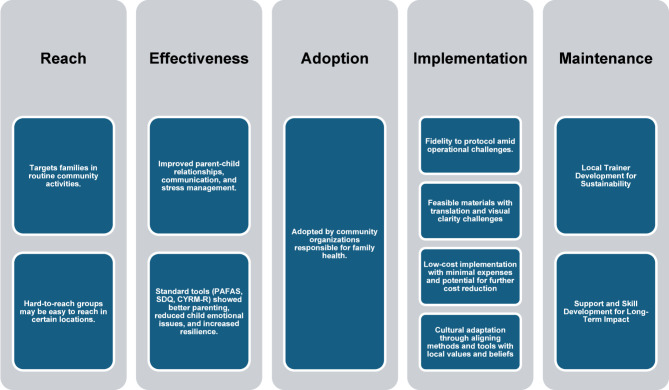
Summarization of Strong Families program using RE-AIM framework

In summary, the program successfully reached families through religious and community organizations, though literacy barriers required verbal communication. Hard-to-reach groups, like those affected by substance abuse, were engaged through rehabilitation centers, while caregivers replaced parents in protective care settings. The program improved parent-child relationships, communication, and stress management, with standard tools (PAFAS, SDQ, CYRM-R) showing positive trends. Adoption was supported by NGOs, religious groups, and the Ministry of Social Development and Human Security of Thailand (MSDHS), with community leaders facilitating expansion. Implementation faced challenges in time, language, and material clarity, but its hands-on approach made it accessible. Adaptations included clearer visuals and cultural modifications. Costs were low, mainly for food and travel. Sustainability focused on training local facilitators, fostering strong relationships, and ensuring structured follow-up (Fig. 3).

## Discussion

The program demonstrated positive outcomes in family strengthening, with improved communication, emotional well-being, and family dynamics. The Strong Families program showed both feasibility and preliminary effectiveness through an evaluation using the RE-AIM framework, as follows.

### Reach

The Strong Families program can reach the population in Thailand’s challenging context through the involvement of community sectors related to child and family care. It relies on strong relationships between civil society, local organizations, and the community through routine activities. Even for hard-to-reach groups, such as those living in orphanages, the program may be easier to access by collaborating with their leaders. This approach is similar to the implementation of the Strong Families program in Serbia and Afghanistan, where good relationships between program organizers and participants contributed to the program’s continuity [16]. Additionally, when participants find that the lessons from the program are practical and applicable, it encourages sustained engagement in program activities [33].

### Effectiveness

We found that the Strong Families program has preliminary effectiveness from both qualitative and quantitative results. The quantitative and qualitative results were in alignment, particularly in terms of the efficacy of the SF program. The quantitative data confirms that these changes occurred in measurable terms across parenting practices, family adjustment, child behavior, and resilience, while the qualitative insights explain how and why changes happened, including learning praise, stress management, and group therapy-like sharing. The Strong Families program’s impact in Thailand is a coherent picture, as both sets of findings support each other. Many countries have been implementing the program [16, 33, 34]. The studies indicated positive feasibility and effect on families’ mental health and parenting skills. Due to the program having sessions for both caregivers and children, therefore it can improve their skills and increase positive factors from both ways [35]. Positive parenting programs offer a broad spectrum of advantages to families, including the cultivation of healthy relationships, the enhancement of communication, and the promotion of stress management [36]. Our results showed that the Strong Families program improved PAFAS by enhancing positive parenting skills. Effective strategies for managing challenging behaviors, establishing boundaries, and reinforcing desirable behaviors in a supportive and non-punitive manner. As a result, the behavioral issues in families would decrease [36]. However, the inconsistent trend in parental consistency scores on PAFAS may be due to each family having more than one caregiver who attended the program in different sessions, as well as the prior relationships between children and caregivers. Regarding the improved SDQ and PMK-CYRM-R, the Strong Families program enhances the mental health and resilience of the entire family by reducing tension and anxiety in both parents and children. Children raised in a positive parenting environment are more likely to excel academically and develop strong social skills, as they feel supported and valued [37]. The flat trend in peer relationship scores on the SDQ may be due to the group of children living in an orphanage. Thus, they face challenges and difficulties in forming new or close relationships with others [38].

### Adoption

This study found that any organization could adopt the Strong Families program, especially those whose workflows align with its objectives, as they often work with families and children. Both public and private sector organizations can integrate the program into their operations. Successful adoption, however, depends on the agency’s readiness, openness, and resistance to change. The program implementation report will offer insights into the factors influencing agency adoption [39]. Furthermore, training should be provided to equip staff with tools to support parenting skills and enhance family health [16].

### Implementation

The Strong Families program is easy to implement and can effectively utilize existing community resources. Consistent with previous findings, the program has proven to be feasible even in resource-limited and conflict settings, such as among Serbian refugees [33]. Parents and caregivers remained engaged because the program addressed their needs for improving family relationships, aligning with previous literature [33]. While these results are positive, different areas may have very different cultural, social, and structural factors that affect how well the Strong Families program works in different situations. These may include community engagement norms, current support systems, and how people think about parenting [40, 41]. In Thailand, community health workers and local government may make it easier for people to use the program. A number of organizations are also working to promote the health of families and children. Parents and caregivers stayed involved in our setting, probably because the program met pressing needs related to family relationships [42]. This is comparatively easier than in conflict settings. This level of involvement might be different in other settings where parenting priorities or challenges are different [43, 44]. However, greater priority is still needed from organizations providing support and services, as well as from policymakers [45].

Since the program manual was developed in other countries in East Asia and the Middle East, some adjustments are necessary to make the materials more relevant to the Thai context, including language and imagery. Ensuring cultural alignment will enhance the program’s effectiveness and ease of implementation [16, 46].

### Maintenance

Sustaining the Strong Families program is essential. Many communities in Thailand and around the world continue to face challenges due to limited awareness of positive parenting skills [47]. Providing support is crucial, particularly by ensuring the availability of trained local personnel to lead and facilitate its implementation. Laypersons in communities have close and strong relationships with the people who live there. Therefore, these connections will enhance the program’s success and engagement. Additionally, support from government and non-governmental organizations remains crucial through coaching, mentoring, and incentive strategies [4, 48].

Many positive parenting programs, such as Parenting for Lifelong Health (PLH) [49] and Preschool Parenting Program (Triple P) [50], have been implemented in Thailand. Compared to these programs, the Strong Families program is simpler, designed for quick implementation in crisis settings, and capable of reaching more families faster. However, its content is not as in-depth as other programs. To enhance its effectiveness, the Strong Families program should incorporate more aspects of parenting and trainer skill development. Another key to sustainability is enhancing trainers’ skills to deal with uncertainty, such as difficult situations (e.g., arguments, anger, silence, or crying participants during activities), which are essential for trainers [51].

The strengths of this study include demonstrating both qualitative and quantitative results from trainers and families, which enhance the feasibility and transferability of the Strong Families program in the Thai context. In addition, we used the standard framework for program evaluation, called “RE-AIM,” throughout the manuscript. This framework highlights essential program dimensions for potential expansion and long-term sustainability. However, there were also limitations. First, the program was evaluated only in the short term, up to six weeks post-training, while its long-term effects are still being assessed. Second, most participants in this study were female. Although this aligns with previous studies, where women were the majority of caregivers or managers in family health fields, it may limit the generalizability of the findings. Third, we did not capture the quantitative results from the children or the perspective from children and their parents, who were the primary target population, due to ethical and reliability concerns. Fourth, we did not collect certain demographic data, such as information on ethnic diversity and caregiving context, which may have limited the depth of our analysis. This limitation should be considered when interpreting the findings. Finally, this is a pilot study. Further research, such as randomized controlled trials, is needed to explore this perspective.

## Conclusion

The program demonstrated positive outcomes in family strengthening, with improved communication, emotional well-being, and family dynamics. Community organizations and religious groups facilitated reach, though challenges included engaging high-risk families and addressing illiteracy. Preliminary effectiveness was supported by improved scores in SDQ and PAFAS metrics. Implementation succeeded with accessible materials and practical activities but faced issues with translations and visuals. Low-cost adoption with cultural adaptations enhanced acceptance. Sustainability focused on local trainer development, strong relationships, and follow-up tools for long-term impact. There is room for improvement in providing more detailed interventions to enhance both parenting and trainer skills. Further randomized controlled trials are needed.

## Data Availability

The datasets used and/or analyzed during the current study are available from the corresponding author on reasonable request.
